# Homogenized modeling methodology for 18650 lithium-ion battery module under large deformation

**DOI:** 10.1371/journal.pone.0181882

**Published:** 2017-07-26

**Authors:** Liang Tang, Jinjie Zhang, Pengle Cheng

**Affiliations:** School of Engineering, Beijing Forestry University, Beijing, China; Chongqing University, CHINA

## Abstract

Effective lithium-ion battery module modeling has become a bottleneck for full-size electric vehicle crash safety numerical simulation. Modeling every single cell in detail would be costly. However, computational accuracy could be lost if the module is modeled by using a simple bulk material or rigid body. To solve this critical engineering problem, a general method to establish a computational homogenized model for the cylindrical battery module is proposed. A single battery cell model is developed and validated through radial compression and bending experiments. To analyze the homogenized mechanical properties of the module, a representative unit cell (RUC) is extracted with the periodic boundary condition applied on it. An elastic–plastic constitutive model is established to describe the computational homogenized model for the module. Two typical packing modes, i.e., cubic dense packing and hexagonal packing for the homogenized equivalent battery module (EBM) model, are targeted for validation compression tests, as well as the models with detailed single cell description. Further, the homogenized EBM model is confirmed to agree reasonably well with the detailed battery module (DBM) model for different packing modes with a length scale of up to 15 × 15 cells and 12% deformation where the short circuit takes place. The suggested homogenized model for battery module makes way for battery module and pack safety evaluation for full-size electric vehicle crashworthiness analysis.

## Introduction

With the strong support from the government [1] and major technology breakthrough for lithium-ion batteries (LIBs) [2, 3], electric vehicles (EVs) have been witnessed to boom over the past recent years [4–6]. The major reason for LIBs to become the primary choice for EVs is due to the combination advantage of high energy/power density, lightweight, and safety [6, 7]. Many research works were conducted on the optimization approach for electrified vehicles to increase cost competitiveness and reduce carbon emissions [6, 8–10]. Additionally, the battery management system plays an important role in maintaining battery lifetime without unduly sacrificing its performance. Some key technologies of battery management system that monitor the unmeasurable internal states of the battery have been extensively studied [11–14]. Battery models such as electrochemical [13], thermal [15], and high-order physics-based model [16] are also used for evaluation of existing charging strategies, estimator and controller development, simulation, and optimization. However, none of these works have considered the mechanical performance of the battery. Because of the inevitable crash or impact for vehicles during traffic accidents, there is a high possibility of internal short-circuit [15], thermal runaway [17], and even explosion [18, 19] for LIBs subjected to external mechanical loading. Thus, the crashworthiness issue of EVs by consideration of LIB safety remains a paramount concern in electric vehicle safety.

In the past years, many pioneering efforts were made to elucidate the mechanical safety behavior of LIBs on multiple length scales, ranging from component material scale to battery pack scale. On the component level, the mechanical properties of the case [20], separator [21], anode, and cathode foils [22] of the cell have been investigated. These results will help to understand the failure mechanism of internal short circuit of lithium-ion battery, but can not characterize the global mechanical behavior of the battery. Many experiments were conducted for the investigation of mechanical behaviors of the battery cells under various loading conditions [20, 23, 24]. The finite element (FE) models were used to understand the mechanical properties and predict extreme cases. Since coating of active material coating and separator are highly porous and soaked in the electrolyte, the detailed modeling including each component and interaction among them is very complicated. Therefore, it is reasonable and acceptable to take jellyroll as a homogeneous material [23, 25, 26]. Furthermore, the dynamic behavior [27] and SOC effect [28] of the battery have been studied and the results suggested that higher SOC leads to higher structure stiffness. In this paper, a computational model of a single cylindrical battery is established and validated based on homogeneous modeling technique.

For battery module and pack, the mechanical safety performance is closely related to sizes and packing modes of the module and modeling every single cell in detail would be costly. Therefore, equivalent homogenized model for the module is fully necessary for many applications such as vehicle level crashworthiness analyses and optimum design. Sahraei et al. [29] used a homogenized crushable foam core to simulate the interior containing battery cells of an idealized battery pack to model the drop test. The battery pack was taken as a linear elastic material in [30] for crash analysis of a conceptual electric vehicle. However, modeling the module by using a simple bulk material would result in sacrificing computational accuracy. Additionally, Lai et al. [31] adopted macro homogenized material models calibrated by the test data to simulate the punch test of a small-scale module specimen. Nevertheless, it is very difficult to directly measure the integrated mechanical properties of the battery module.

This paper responds to the challenge by extracting a representative unit cell (RUC) with the periodic boundary condition applied on it to analyze the homogenized mechanical properties of the module. An elastic–plastic constitutive model is established to describe the equivalent battery module (EBM) model. Further, a small-scale battery module is tested to compare the mechanical behavior with those obtained from the EBM model and detailed battery module (DBM) model. Upon the satisfactory comparison results, EBM model is further generalized for larger battery modules and different packing modes and the feasibility of the established model is discussed.

## Methods

### 2.1 Computational model of a single cell LIB

**Fig 1:**
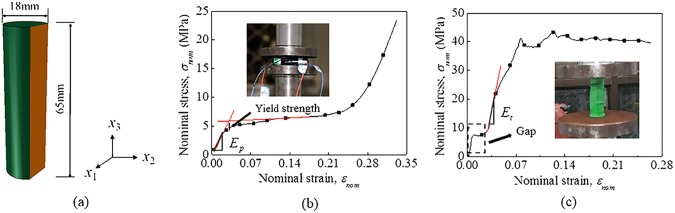
Mechanical behavior of 18650 lithium-ion battery. (a) The 18650 lithium-ion battery. Nominal stress–strain curve of a single LIB under (b) radial compression and (c) axial compression conditions.

Mechanical behavior of a single cell LIB surely dominates that of a battery module. The 18650 LixC6/LiCoO2 batteries are used in this study, provided by SONY. The main cell geometry and dimensions are provided in Fig 1A. The cylindrical LIB cell is composed of winding cathode, anode, and separator films and encapsulated into a steel can. So it is extremely complicated to simulate each component and interaction among them in detail. Thus, the entire LIB is taken as an orthotropic homogeneous material. For simplicity, the *x*1—*x*_2_ plane that is perpendicular to the axis of the battery is considered as isotropic and a transversely isotropic elastic plastic model has been adopted to express the mechanical properties.

The stress-strain laws for transversely isotropic elasticity are shown as follows:

$$\left\{\begin{array}{l} \varepsilon_{11}\\ \varepsilon_{22}\\ \varepsilon_{33}\\ \gamma_{12}\\ \gamma_{13}\\ \gamma_{23} \end{array}\right\}=\left[\begin{array}{cccccc} 1/E_{p} & -\nu_{p}/E_{p} & -\nu_{tp}/E_{t} & 0 & 0 & 0\\ -\nu_{p}/E_{p} & 1/E_{p} & -\nu_{tp}/E_{t} & 0 & 0 & 0\\ -\nu_{pt}/E_{p} & -\nu_{pt}/E_{p} & 1/E_{t} & 0 & 0 & 0\\ 0 & 0 & 0 & 1/G_{p} & 0 & 0\\ 0 & 0 & 0 & 0 & 1/G_{t} & 0\\ 0 & 0 & 0 & 0 & 0 & 1/G_{t} \end{array}\right]\left\{\begin{array}{l} \sigma_{11}\\ \sigma_{22}\\ \sigma_{33}\\ \sigma_{12}\\ \sigma_{13}\\ \sigma_{23} \end{array}\right\}$$ (1)

where *p* and *t* stand for “in-plane” and “transverse,” respectively. Thus, *E*_*p*_ and *E*_*t*_ are Young’s modulus of radial and axial directions, *E*_1_ = *E*_2_ = *Ep* and *E*_3_ = *E*_*t*_; *G*_*p*_ and *G*_*t*_ are the shear modulus of the corresponding planes, *G*_12_ = *G*_*p*_ and *G*_13_ = *G*_23_ = *G*_*t*_; *ν*_*p*_, *ν*_*tp*_ and *ν*_*pt*_ are the Poisson’s ratio, *ν*_12_ = *ν*_*p*_ and *ν*_13_ = *ν*_23_ = *ν*_*pt*_; and subscripts “1”, “2” and “3” stand for the coordinate system.

The anisotropic yield behavior is modeled through yield stress ratio, *R*_*ij*_, which is applied in Hill’s potential function. The yield stress ratio is defined as follows:

$$R_{ij}=\left[\begin{array}{ccc} \frac{{\bar{\sigma }}_{11}}{\sigma_{0}} & \frac{{\bar{\sigma }}_{12}}{\tau_{0}} & \frac{{\bar{\sigma }}_{13}}{\tau_{0}}\\ & \frac{{\bar{\sigma }}_{22}}{\sigma_{0}} & \frac{{\bar{\sigma }}_{23}}{\tau_{0}}\\ & & \frac{{\bar{\sigma }}_{33}}{\sigma_{0}} \end{array}\right]$$ (2)

where each ${\overset{-}{\sigma }}_{ij}$ is the measured yield stress value; *σ*_0_ is the user-defined reference yield stress set as 5 MPa; $\tau_{0}=\sigma_{0}/\sqrt{3}$.

The plastic-hardening model for the compression is expressed as the form of the Eq. (3) proposed by Greve and Fehrenbach [23]:

$$\sigma =A\varepsilon^{n}+B$$ (3)

where *A* and *B* are the parameters to be determined, *σ* is the stress, *ε* is the plastic strain, and *n* is the hardening exponent.

The above developed mechanical model is calibrated through the radial compression and axial compression tests, as shown in Fig 1B and 1C. The compression tests were conducted on INSTRON 8801 platform with a loading rate of 2 mm/min, where the indenter and the bearing were both flats. The force and displacement sensors were set on the indenter. For radial compression, the nominal stress-strain curve can be converted from the load-displacement curve as follows:

$$\sigma_{\text{nom}}=\frac{\text{F}}{S_{c}}$$ (4)

$$S_{c}=l_{c}b_{c}$$ (5)

$$b_{c}=2R\arccos(\frac{R-s/2}{R})$$ (6)

$$\varepsilon_{\text{nom}}=\frac{s}{2R}$$ (7)

where *F* is the load and *S*_*c*_ stands for the contact area; *l*_*c*_ is the length of the cell and *b*_*c*_ is the contact width; R is the radius of the cell and *s* is the displacement of the indenter. The radial compressive nominal stress–strain curve distinctly has three stages, as follows: linear region, plateau region, and densification region. The axial modulus is extracted from the second stage of the curve because the cap collapsed during the axial compression test. The parameter values for the mechanical model of a single cell are summarized in Table 1.

**Table 1:** Summary of material properties for a single cell.

Elasticity modulus	Poisson’s ratio	Yield stress ratios	Hardening model
$\begin{array}{l} E_{p}=260MPa\\ E_{t}=1200MPa\\ G_{p}=118MPa\\ G_{t}=500MPa \end{array}$	$\begin{array}{l} \nu_{\text{p}}=0.1\\ \nu_{pt}=0.08 \end{array}$	$R_{ij}=\left[\begin{array}{ccc} 1.1 & 1.8 & 12\\ 0 & 1.1 & 12\\ 0 & 0 & 8.4 \end{array}\right]$	$\begin{array}{l} A=19000\\ B=5.83\\ n=5.9 \end{array}$

**Fig 2:**
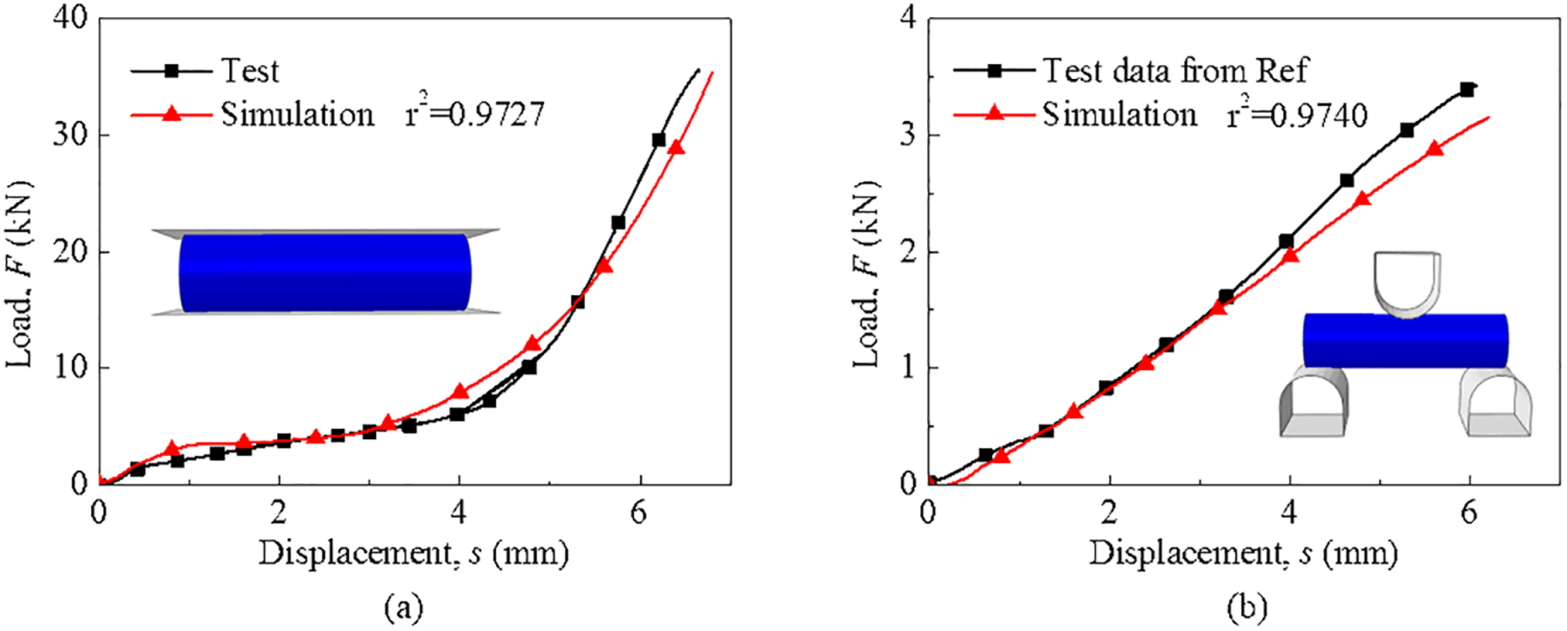
Validation for the computational model of 18650 lithium-ion battery. Mechanical responses from tests and simulations in loading conditions: (a) radial compression and (b) bending.

To validate the model, the simulations of radial compression and bending were conducted based on the ABAQUS/Explicit platform on which the proposed constitutive model was used. The bending experiment data were cited from open literature and more detailed information could have been referred from another study [26]. The computational model of the 18650 lithium-ion battery cell was composed of eight-node linear brick, reduced integration solid element with the size of 0.8 mm. As illustrated in Fig 2A and 2B, the model can well predict the mechanical behavior of the cell under radial compression and bending loading conditions, where the coefficient of determination $r^{2}=1-\frac{\sum {\left(y_{i}-f_{i}\right)}^{2}}{\sum {\left(y_{i}-\overset{-}{y}\right)}^{2}}=0.97$ (where *y*_*i*_ and *f*_*i*_ are the experimental and simulated data, respectively, and $\overset{-}{y}$ is the average of the experimental data).

### 2.2 RUC extraction for the battery module

**Fig 3:**
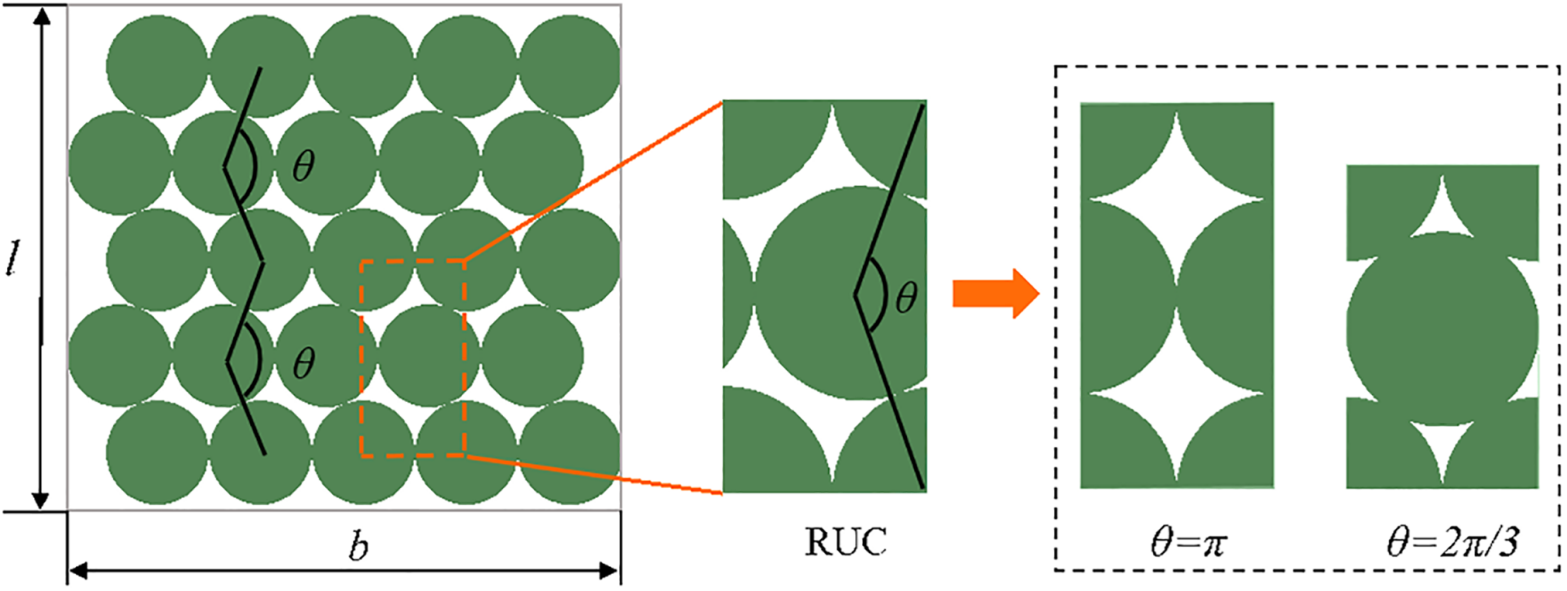
Battery module and extraction of RUC. Diagram of battery module and RUC defined by *l*, *b*, and *θ* and typical RUCs with *θ* = *π* and 2*π*/3.

The packing method of cylindrical cells can be treated as a geometrical model, where packing sizes and modes of the module were uniformly defined with *b*, *l*, and *θ* as shown in Fig 3. *b* and *l* are the number of cells on each row and column and *θ* describes the relative position of rows, which varies from 2*π*/3 to *π*. The packing density of the module can be expressed as follows:

$$\rho =\frac{\pi bl}{4(b+\cos\frac{\theta }{2})(l\sin\frac{\theta }{2}+1-\sin\frac{\theta }{2})}$$ (8)

Since the battery module can be regarded as the periodic arrangement of RUCs, RUC of the packing module is extracted. As shown in Fig 2, RUC is related to *θ*, where the nominal density should be calculated as the following:

$$\rho_{\text{RUC}}=\frac{\pi }{4\sin\frac{\theta }{2}}(\frac{2\pi }{3}<\theta \leq \pi )$$ (9)

Naturally, *ρ*_RUC_ equals the packing density of a module when *b* and *l* are regarded as infinite. In this paper, the mechanical properties of RUC are assumed to be equivalent to the properties of the module. When *θ* = *π* and *θ* = 2*π*/3, i.e., cubic dense packing and hexagonal dense packing, the RUC is symmetrical in structure, and the structure of module is stable during compression deformation.

### 2.3 Computational model for RUC

**Fig 4:**
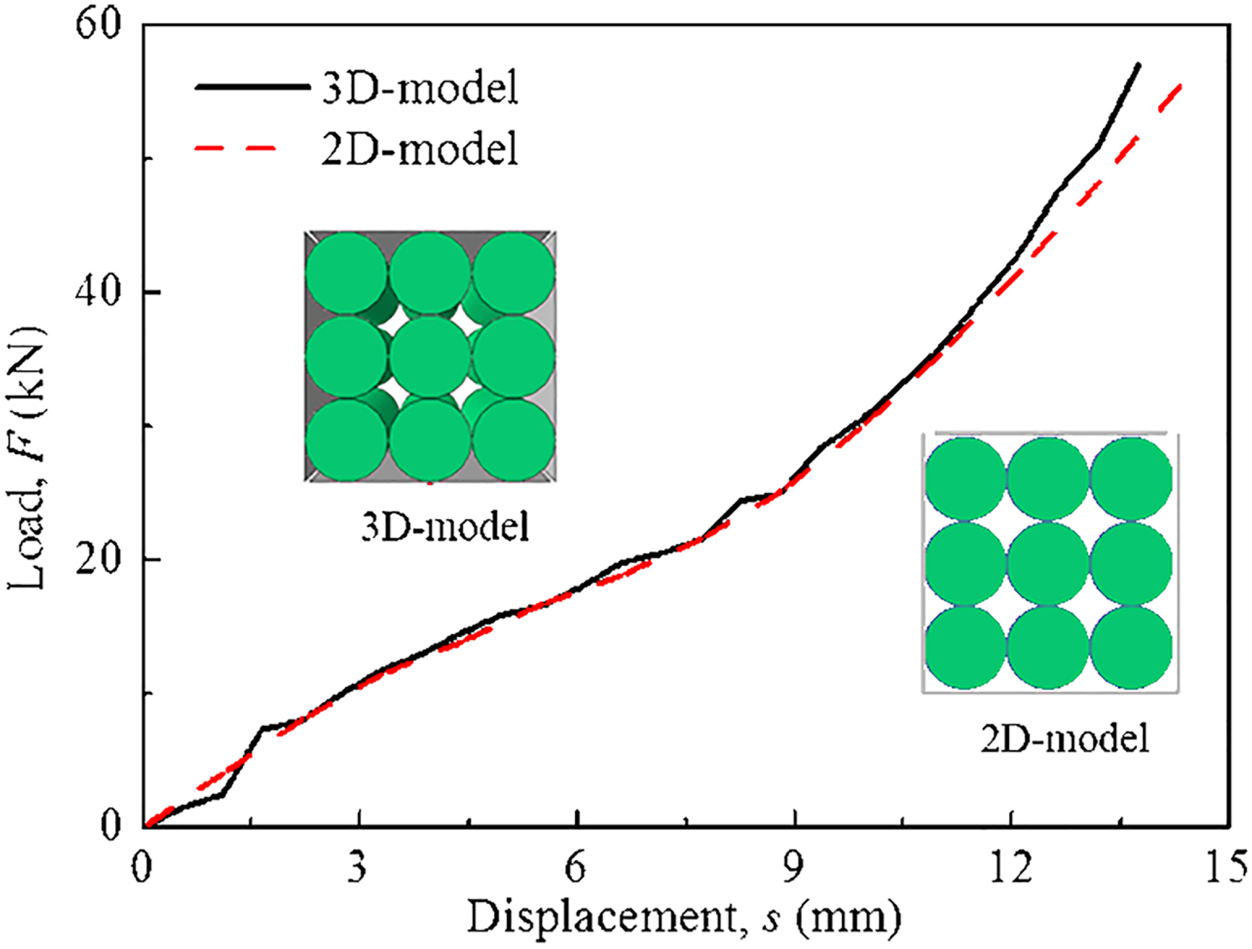
Comparison of 3D and 2D models under constrained compression loading conditions.

For a battery module, the length of the cell is sufficiently long relative to its axial deformation, and every cross section of the module perpendicular to the axes of the cell is identical. Therefore, it is reasonable to be simplified into a two-dimensional plane strain problem. In order to verify the simplification from 3D to 2D, constrained compression simulations using 3D model and 2D model, respectively, were performed, as shown in Fig 4. The results of 2D model and 3D model are almost the same with each other. Then, 2D model is chosen for the next works.

2D model was developed to represent the 3D RUC and the RUC FE model was composed of four-node bilinear quadrilateral plane strain, which reduced integration 2D solid elements with the size of 0.8 × 0.8 mm. A penalty based contact was set for the contacting parts of adjacent cells by reasonably assuming a friction coefficient of 0.1. To understand the compressive mechanical properties of RUC, a periodic boundary condition that has been investigated in Ref [32–35] was applied to the boundaries of RUC and the boundary equation, as follows:

$$u_{i}^{j+}-u_{i}^{j-}={\bar{\varepsilon }}_{ik}\Delta x_{k}^{j}(i,j=1,2,3)$$ (10)

where *u* is the displacement of note, ${\overset{-}{\varepsilon }}_{ik}$ are the average strains, index ‘‘*j+*” means along the positive *X*_*j*_ direction and ‘‘*j−*” means along the negative *X*_*j*_ direction and $\Delta x_{k}^{j}$ are distances between pairs of opposite boundary surfaces. The top and bottom sides were forced and fixed separately to achieve the mechanical properties.

### 2.4 Homogenized method for battery module modeling

A homogenized method is proposed to replace the detailed modeling method for the battery module. The mechanical properties of the homogenized EBM model can be equivalent to the properties of the RUC model.

**Fig 5:**
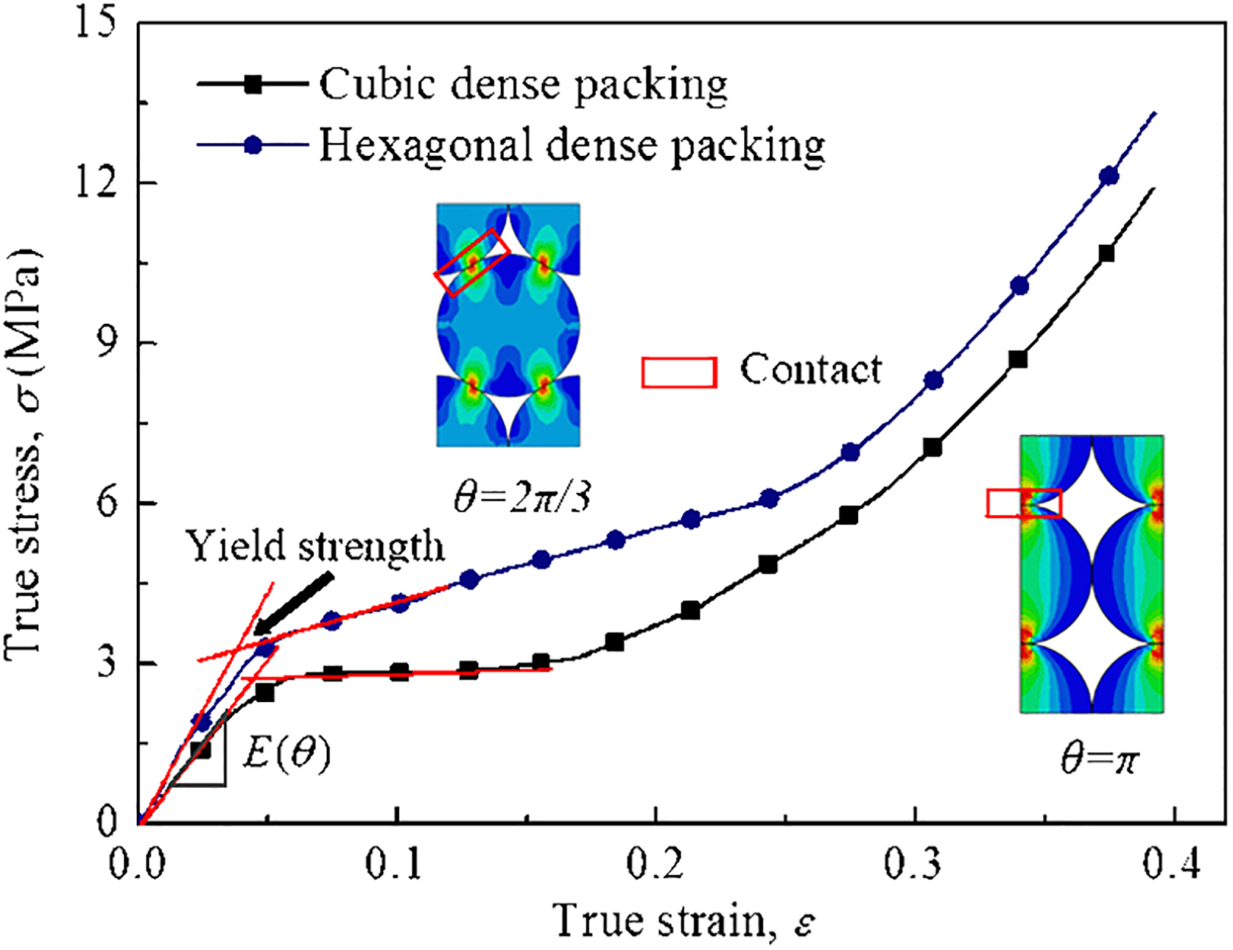
Mechanical properties of RUC. True stress–strain curves of two typical RUCs in uniaxial compression simulation. The plots of deformed RUCs are shown in subplot of the figure.

Fig 5 shows the true stress–strain curves of RUCs for two typical packing modes during uniaxial compression deformation. The true stress–strain curve could be converted from the nominal stress–strain curve as follows:

$$\begin{array}{l} \varepsilon_{\text{true}}=\ln(1+\varepsilon_{\text{nom}})\\ \sigma_{\text{true}}=\sigma_{\text{nom}}(1+\varepsilon_{\text{nom}}) \end{array}$$ (11)

where *ε*_nom_ and *σ*_nom_ can be calculated from the load force and displacement divided by the contact area and the original height of the RUC. The curves first increase linearly to a plateau stage and then increase sharply after the plateau stage. Apparently, there is a difference between the two curves that the stress for hexagonal dense packing is higher than that of cubic dense packing because of its larger density.

An elastic–plastic material model can be used to predict the homogenized EBM behavior. Young’s modulus *E* (*θ* = *π*) and *E* (*θ* = 2*π*/3) are calculated from above true stress–strain curves as 55 and 90 MPa, respectively. The plastic stage of the curves are used as input file of the plastic part of the material model.

### 2.5 Experiment setups for EBM model verification

**Fig 6:**
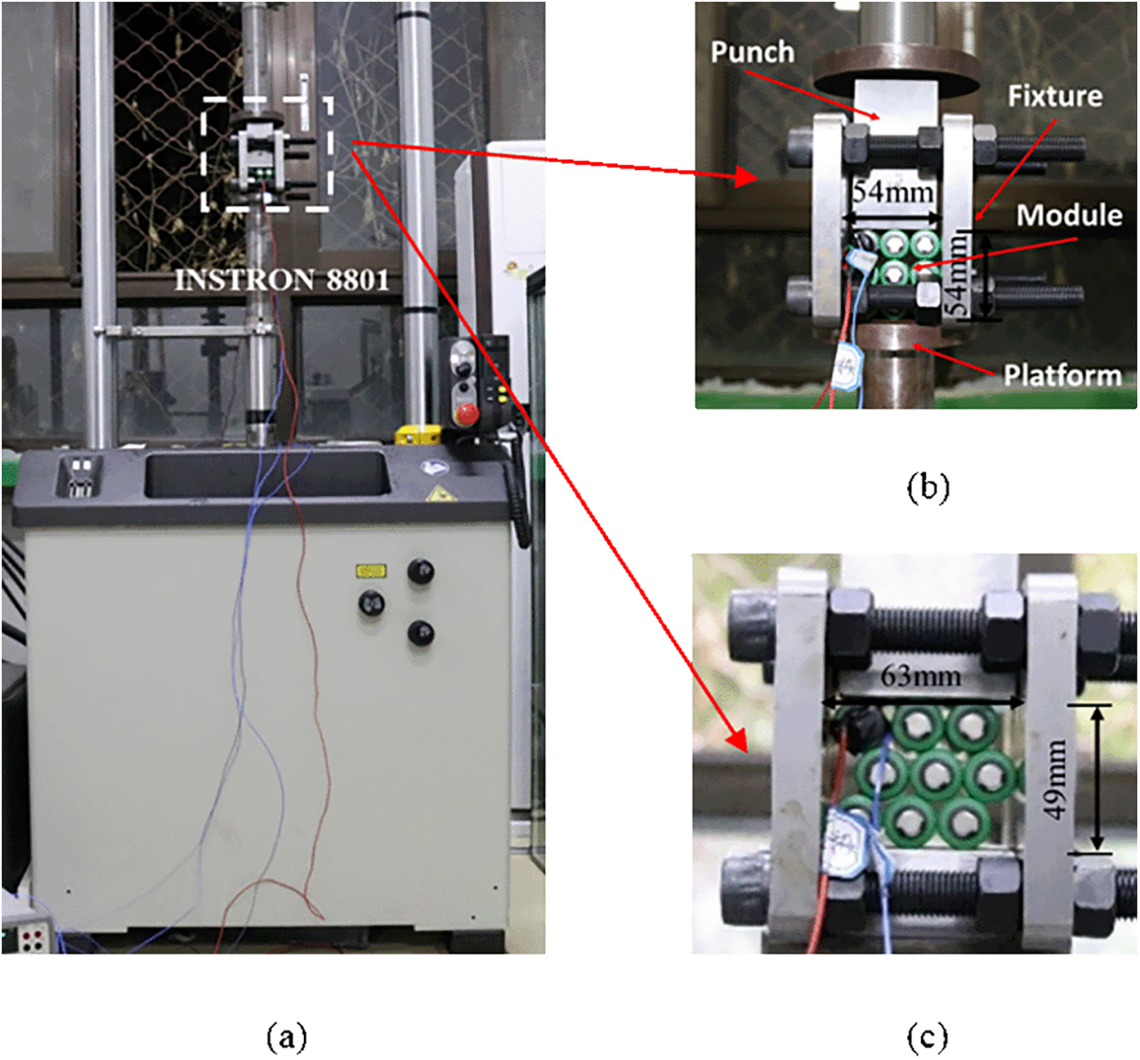
Experiment setups for validation. (a) The setups of compression experiments for the module. Battery module for (b) cubic dense packing and (c) hexagonal dense packing during compression experiment.

To validate the proposed homogenized EBM model, the constrained compression experiments in quasi-static loading condition that commonly occurred during traffic accidents were conducted on INSTRON 8801 platform with a loading rate of 2 mm/min as shown in Fig 6. The battery modules included nine fully discharged battery cells, which are in contact with one another for typical cubic dense packing and hexagonal dense packing. Connection tabs and cooling systems existing in a realistic battery module were neglected. The width and height of the module were within 65 mm in a small size. Moreover, the battery module was restrained by two steel plates with thickness of 20 mm. The distance between the two plates could be adjusted to the size of the module and the plates are fixed by bolts. For reduction of friction effect, the width of load transfer punch is slightly smaller than the distance between the plates. The battery module was compressed by 10 mm, which was about 20% of its height.

## Results

Experiments, homogenized EBM model and corresponding DBM model were conducted on the case for the cubic dense packing mode, i.e. *θ* = *π*. The settings of boundary conditions for both computational models were exactly the same as in the experiment.

For the EBM computational model, four-node bilinear quadrilateral plane strain, which reduced the integration of 2D solid elements, was used; this was also verified via convergence study. Moreover, the proposed homogenized mechanical properties in Section 2.4 were adopted. For the DBM model, nine single battery cell models were included that developed in Section 2.1. The supporting platform and the indenter were set as discrete rigid. The indenter was designated a certain displacement with quasi-static compressive loading. A penalty-based contact was set for the contacting parts by reasonably assuming a friction coefficient of 0.1.

**Fig 7:**
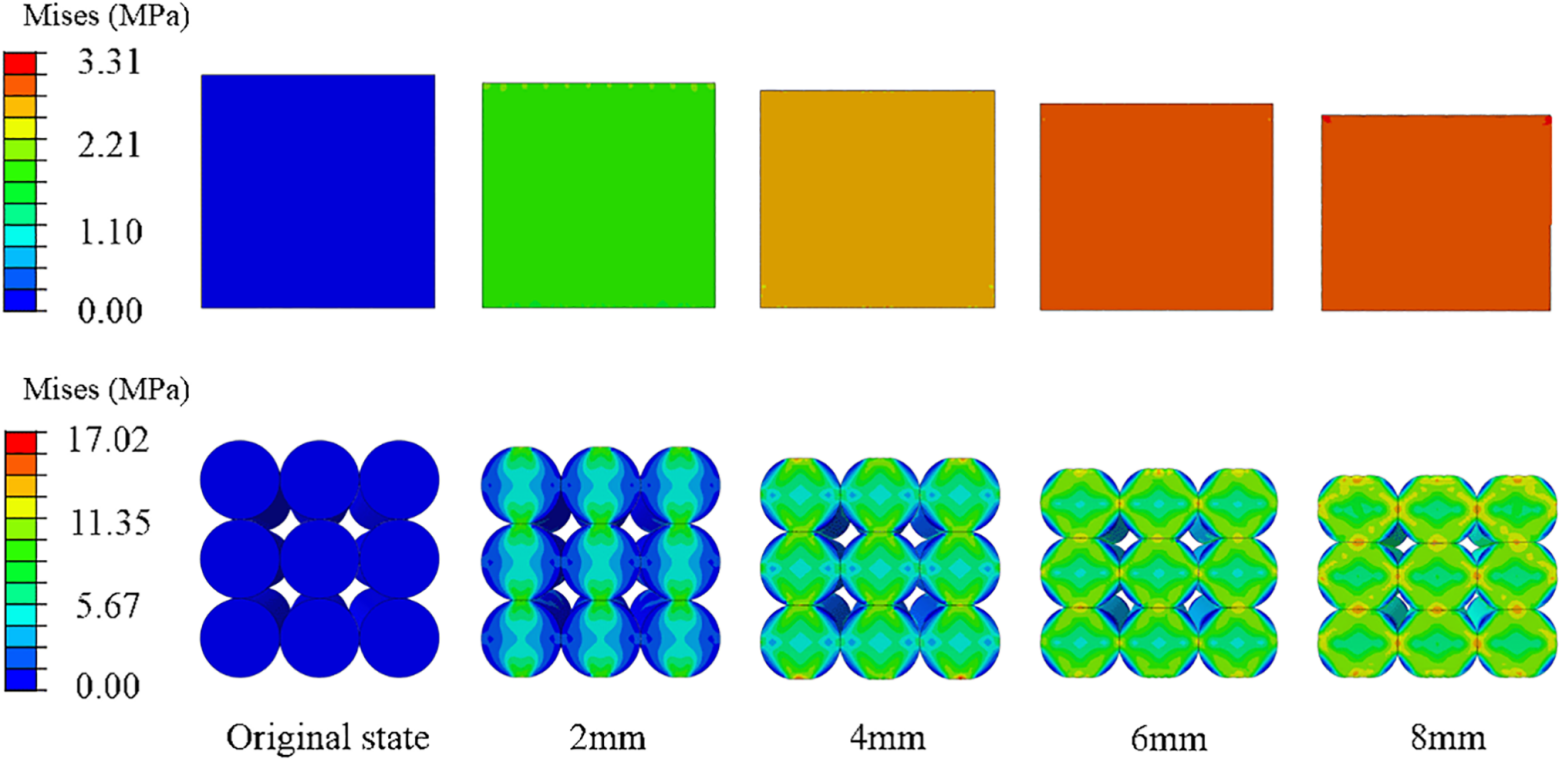
The stress evolution of the module during deformation. Progression of the buildup under constrained compression condition for EBM model (top) and DBM model (bottom).

The stress evolution of the module during deformation is shown in Fig 7. The stress distribution of EBM model is mean, and the corners near the load are under high stress state. For the DBM model, the stress wave spreads from contact area along the load direction to the rest part of the cell, and the stress values of contacting areas of the neighboring cells are higher. In addition, the maximum stress value of the EBM model is smaller than that of DBM model under the same deformation because of larger contact area. It should be noted that, as the deformation increases, the stress growth of the EBM model slows down. As presented in the subsequent section, in the overall mechanical response, the deviation of the load between the EBM model and the DBM model will increase dramatically when the deformation exceeds a certain range. Therefore, the homogenized EBM model can only precisely predict the mechanical behavior of a battery module within a certain deformation range.

**Fig 8:**
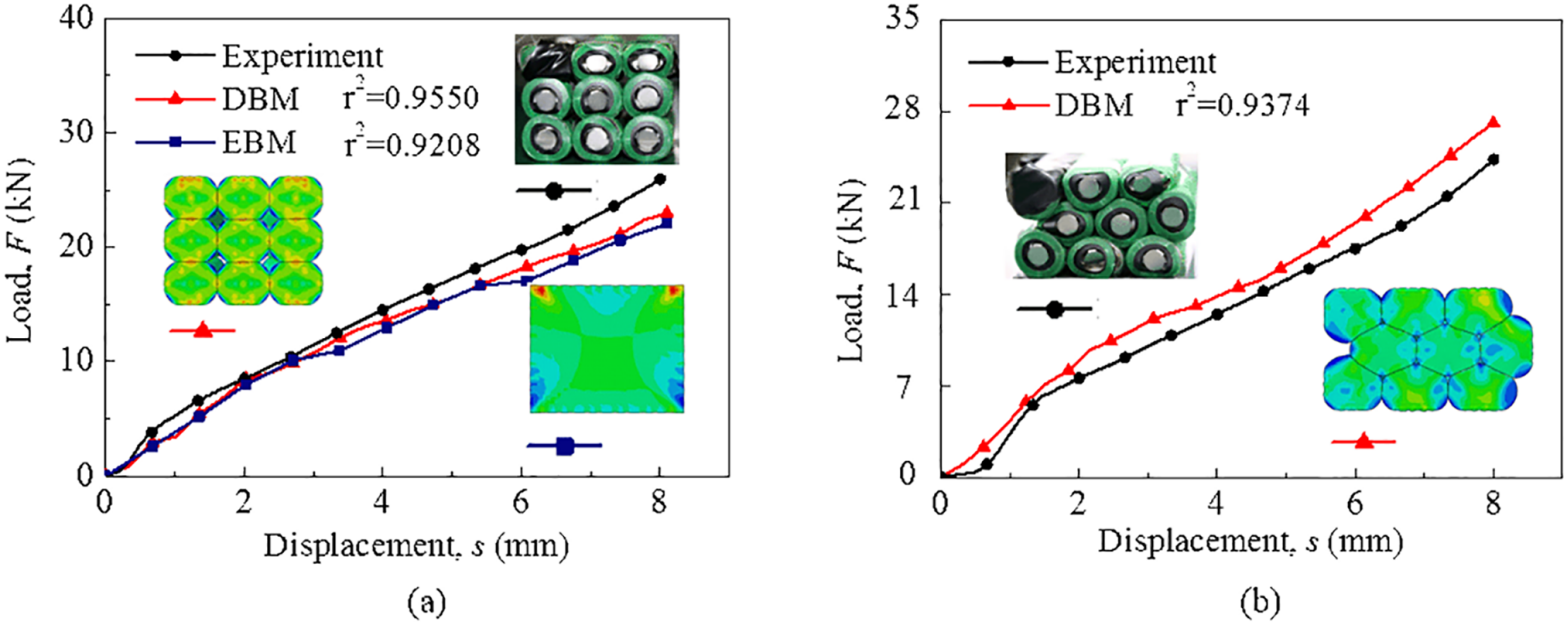
Mechanical responses of the battery module in small size. Comparison of results of the module under constrained compression condition for (a) cubic dense packing and (b) hexagonal dense packing. The plots of deformed modules in the experiment and simulations are shown in the subplot of the figure.

Comparison of the mechanical responses of the battery module for cubic dense packing is shown in Fig 8A. The load-displacement curves are close to one another (where r^2^ = 0.92–0.96), thereby indicating that the EBM model can well predict the integral mechanical behavior of the battery module under constrained compression loading conditions on a small size. It is noted that the EBM model could not distinctively simulate the local mechanical behavior such as contacts between neighboring cells.

Additionally, as illustrated in Fig 8, the DBM model can well reflect the mechanical behavior and deformation of the module for cubic dense packing (where r^2^ = 0.96) and hexagonal dense packing (where r^2^ = 0.94), so it could be used as a method to validate the EBM model if it is difficult to conduct an experiment.

## Discussions

### 4.1 Applicability for the battery module under large size

In general, there are more than nine cells in a realistic battery module. Therefore, the applicability of the homogenized modeling method for the module should be validated under large size. The EBM model and DBM model under different sizes were conducted in constrained compression loading condition for two typical packing modes, i.e., cubic dense packing and hexagonal dense packing. Because the maximum force generated during experiments for the module including nine cells already reached almost 100 KN load limit of the INSTRON 8801, the validation under larger size will be conducted by the DBM model.

**Fig 9:**
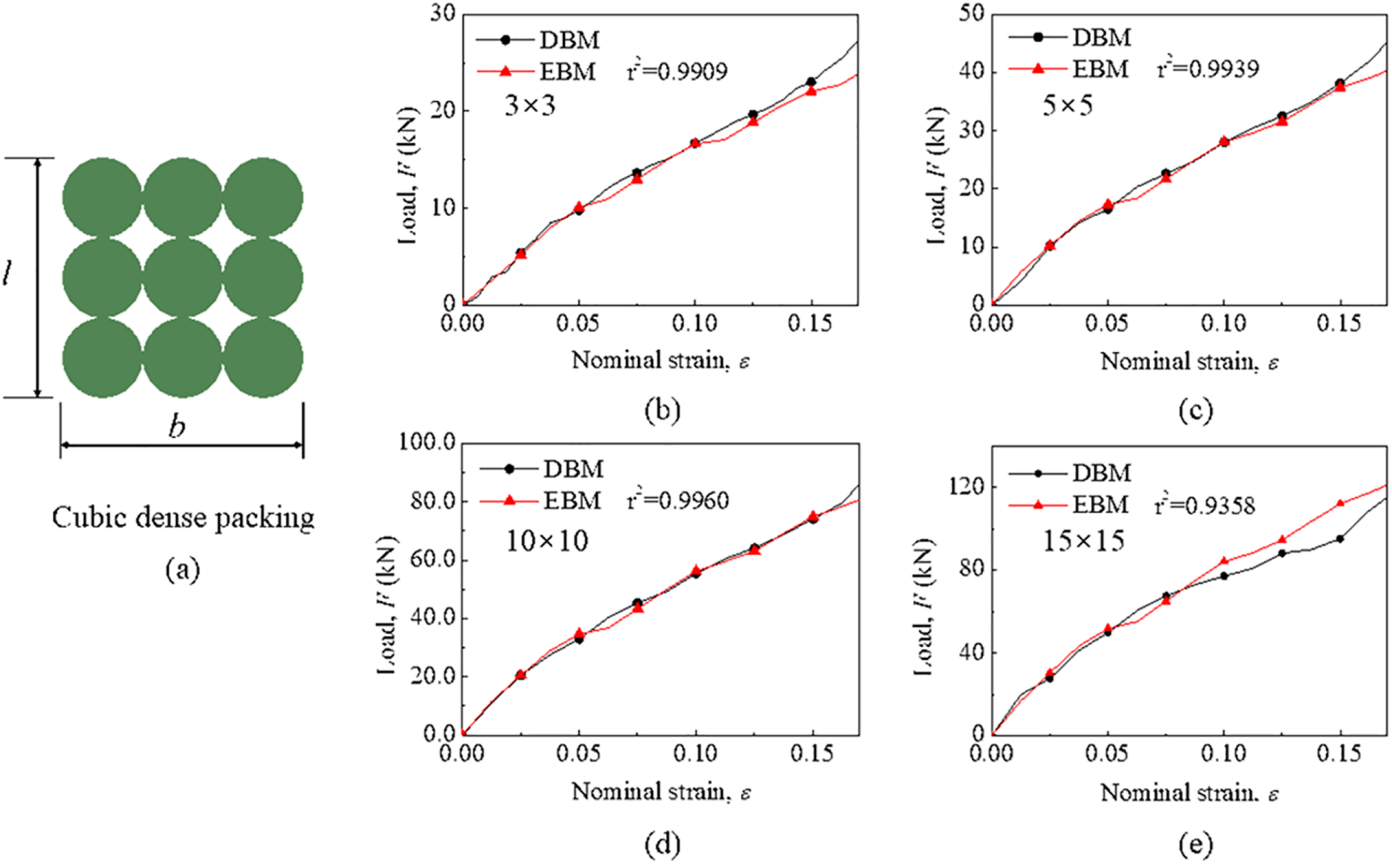
Validation for cubic dense packing under different sizes. (a) Size of cubic dense packing defined by *b* and *l*. Comparisons of mechanical responses of the module under different sizes between the EBM model and DBM model where the packing density is constant. The sizes of the module are labeled in each graph.

For the cubic dense packing mode, the packing density of a module is equivalent to that of corresponding RUC under different sizes. Fig 9 shows the comparisons of mechanical responses between the EBM model and DBM model under different sizes. For observation convenience, the mechanical responses are expressed as the form of load–strain curves and the subscript “*b* × *l*” denotes that a number of *b* and *l* cells are placed in each row and column, respectively. It is found that there is a good agreement between the EBM model and DBM model under each size (where r^2^ = 0.94–0.99), which indicates that the mechanical behavior can be predicted well by this model with length scale up to 15 × 15 in the range of 15% deformation where the structure of cubic dense packing is stable. When the size reaches a greatly extent, the slight dislocation of individual cells in the module will occur due to deformation of cells, which leads to a load decline.

**Fig 10:**
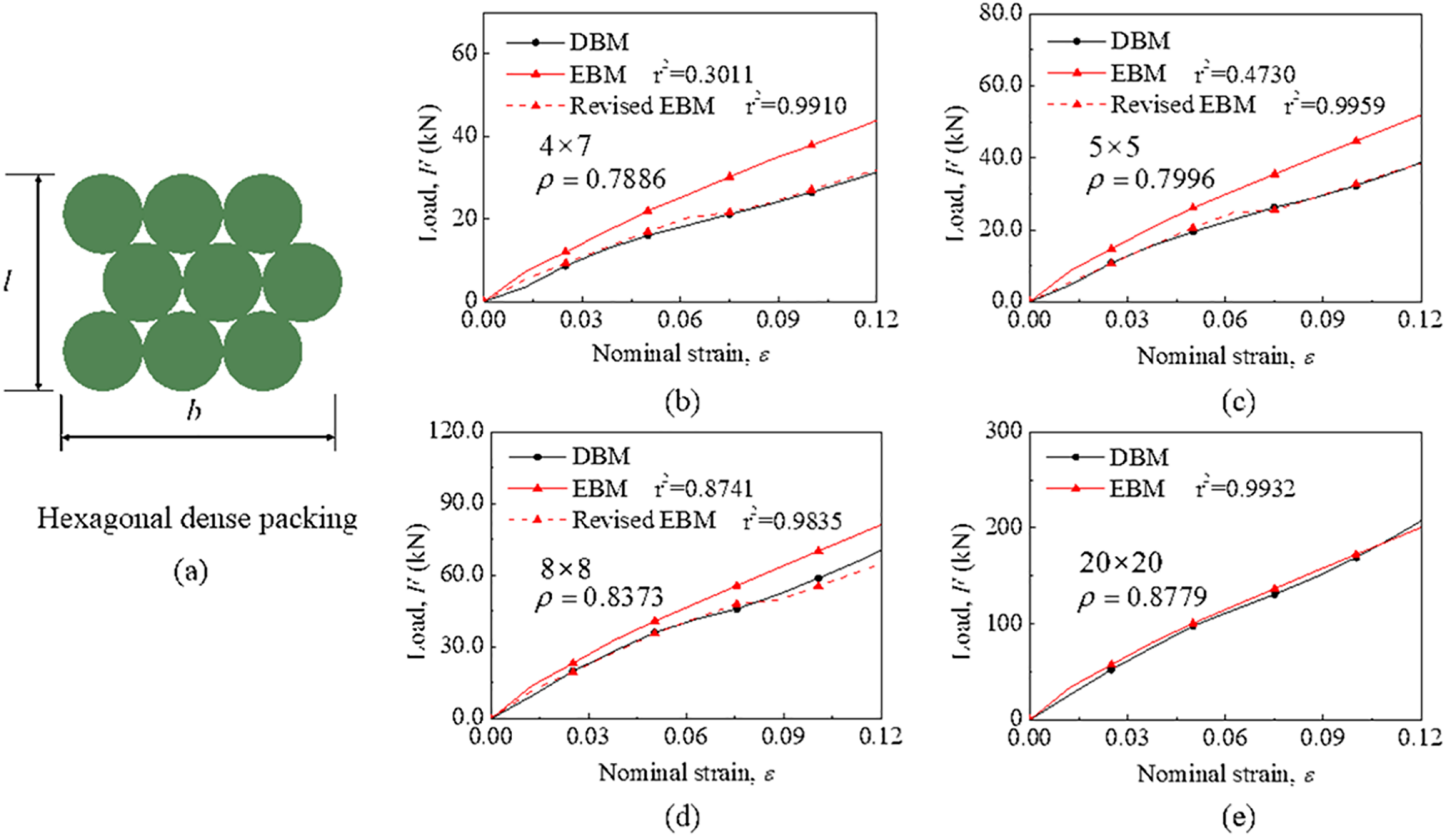
Validation for hexagonal dense packing under different sizes. (a) Size of hexagonal dense packing defined by *b* and *l*. Comparisons of mechanical responses of the module under different sizes between the EBM model and DBM model where the packing density is variable. The sizes and densities of the module are labeled in each graph.

For the hexagonal dense packing mode, the packing density of a module increases as the increase of its size *b* and *l* from Eq. (8). Fig 10 shows the comparisons of mechanical responses between the EBM model and DBM model as well as the packing density of the module under different sizes. It is observed that the difference between the EBM model and DBM model becomes small gradually as the size increases (where r^2^ increases from 0.30 to 0.99). Since the effect of size could be eliminated according to the aforementioned result of cubic dense packing, it is reasonable that the EBM model can more precisely describe the module when the density of the module approaches that of its RUC.

**Fig 11:**
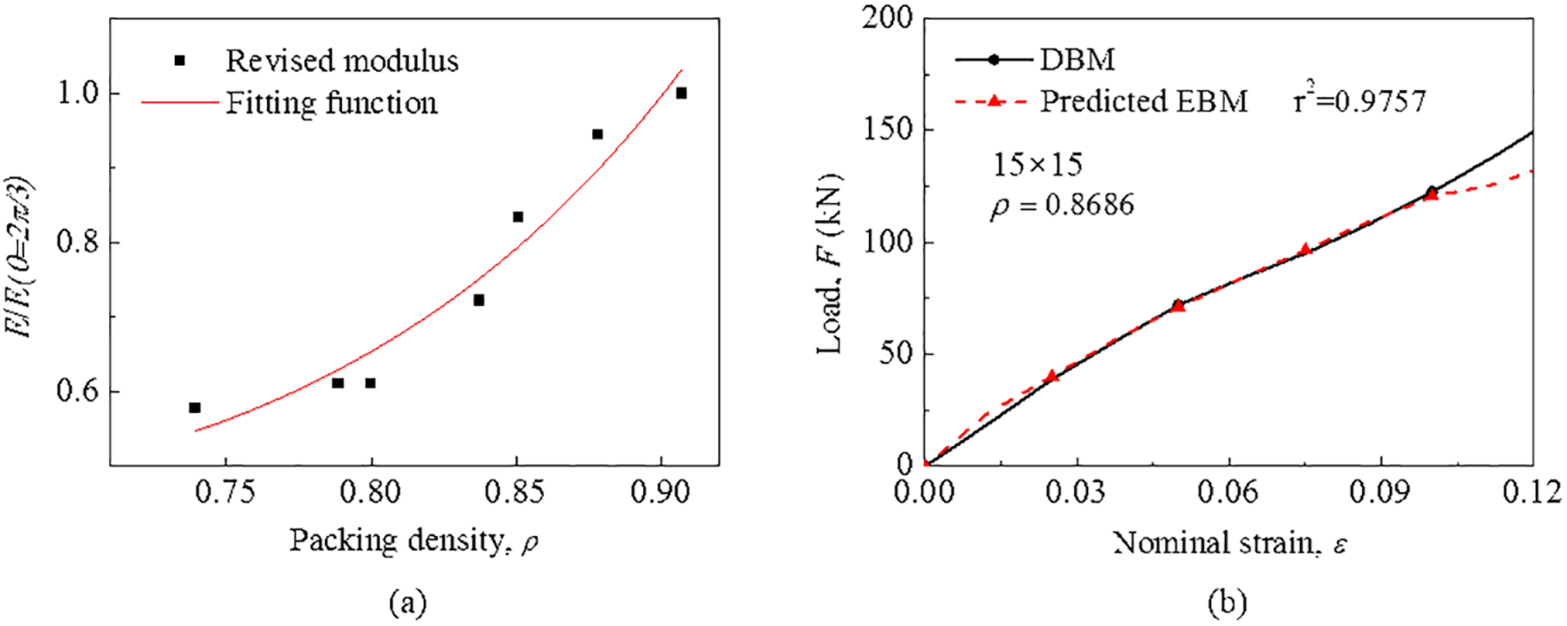
The revised EBM model for hexagonal dense packing. (a) The function relationship with packing density for hexagonal dense packing. (b) Comparison of mechanical responses of the module between the predicted EBM model and DBM model.

When the battery module is taken as a homogenized composite where cells and air are seen as fibers and matrix, respectively, its elastic modulus depends on the volume fraction and elastic property of components according to the composite rule of mixture. Therefore, the elastic modulus of the EBM model can be modified to make this model more accurately describe the module under different sizes. Fig 10 shows that the revised EBM model can predict well the mechanical behavior under different sizes in the range of 12% deformation (where r^2^ = 0.98–0.99). The ratios of modified elastic modulus *E* and original modulus *E* (*θ* = 2*π*/3) are shown in Fig 11A and fitted as a function relationship with packing density *ρ* as follows:

$$\frac{E}{E(\theta =2\pi /3)}=1.29\rho^{7.6}+0.42$$ (12)

As shown in Fig 11B, as predicted by Eq. (12), the EBM model can reflect well the mechanical behavior of the module for hexagonal dense packing under “15 × 15” size with r^2^ = 0.98.

### 4.2 Applicability for different packing modes

**Fig 12:**
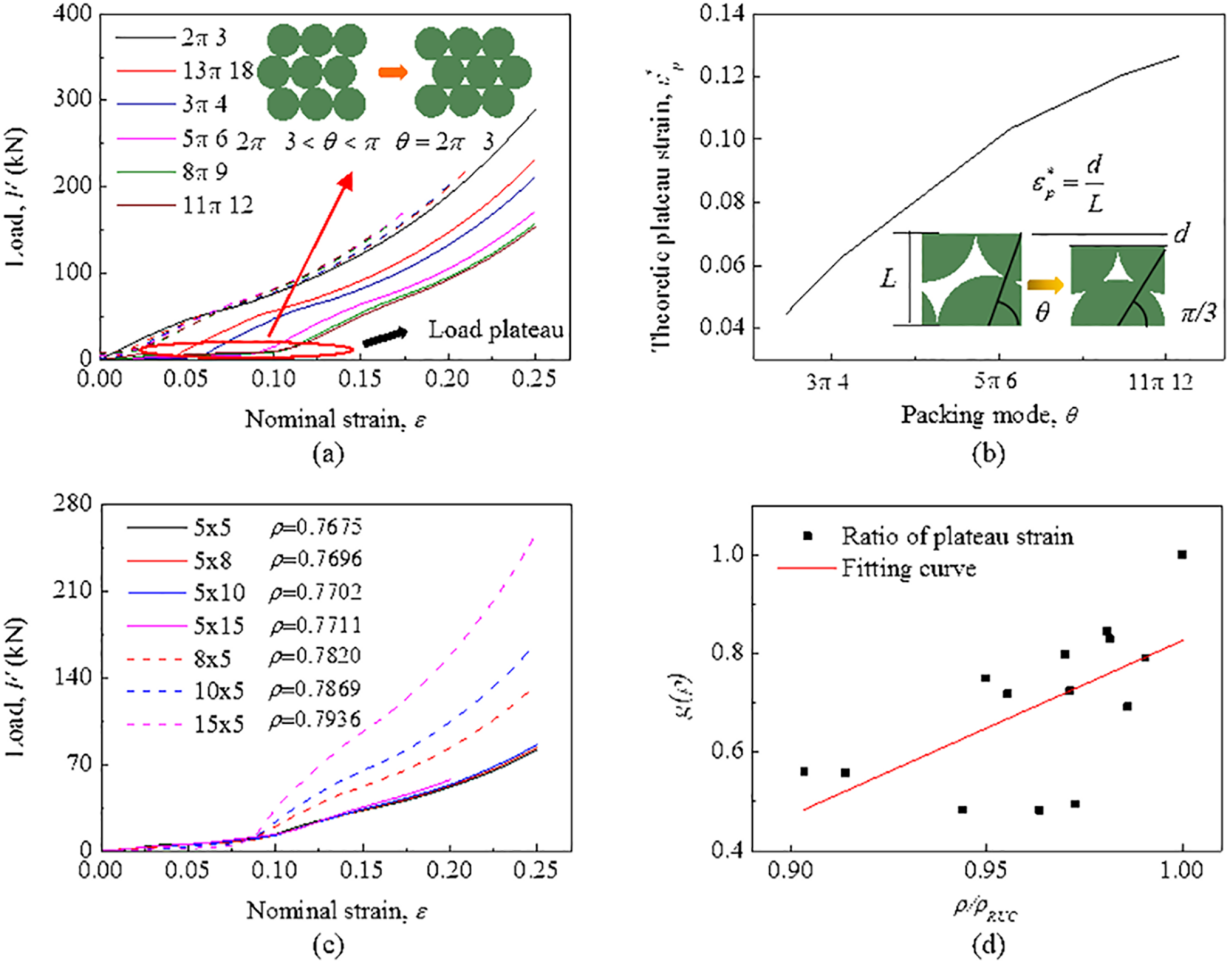
Mechanical behavior of the module for asymmetric packing modes. (a) Mechanical responses of the module with a DBM model for different packing modes when *b* = l = 10. (b) The relationship of theoretical plateau strain and packing mode. (c) Mechanical responses of the module for *θ* = 5π/6 with variables *b* and *l*. (d) The fitting function relationship with the ratio of packing density to that of corresponding RUC.

Besides aforementioned two typical packing modes where the structure of the module is stable during compression deformation, the cylindrical battery cells can also be arranged for other packing modes, i.e., 2*π*/3 < *θ* < *π*. Fig 12A shows the mechanical behavior of modules with the same size (“10 × 10”) for different packing modes. The load plateau can be observed, which is negligible in the whole compression process. After removing the load plateau, the remainder of the curve is parallel to the curve that describes the module for hexagonal dense packing (the dotted lines represent the remainder of the curves). Evidently, the reason for this phenomenon is that the individual cells in the module overcome the friction to conduct relative motion and tend to form hexagonal dense packing. Thus, the verified EBM model for the hexagonal dense packing mode can be used to predict the mechanical behavior for the asymmetric packing modes, only removing the load plateau stage.

As demonstrated in Fig 12B, when the module fully converts to the hexagonal dense packing mode, assuming the cell was not deformed, the theoretical plateau strain $\varepsilon_{p}^{*}$ can be calculated through the geometrical relationship as follows:

$$\varepsilon_{p}^{*}=1-\frac{\sqrt{3}}{2\sin\frac{\theta }{2}}$$ (13)

The equation shows that the length of the load plateau increases with the augment of *θ*, and that is in accord with what is presented in Fig 12A. In fact, the plateau length is always smaller than the theoretical value because of the limit of size and boundary condition. Fig 12C shows the mechanical responses of the module for *θ* = 5π/6 with variables *b* and *l*. It is suggested that the plateau length hardly depends on the size while closely related to packing density. An empirical formula predicting the length of plateau can be proposed as follows:

$$\varepsilon_{p}=f(\theta ,\rho )=\varepsilon_{p}^{*}(\theta )g(\rho )$$ (14)

As shown in Fig 12D, since the range of packing density of a module relies on its packing mode, *g*(*ρ*) can be fitted as the following:

$$g(\rho )=3.54\frac{\rho }{\rho_{\text{RUC}}}-2.71$$ (15)

where *ρ*_RUC_ is the density of RUC calculated from Eq. (9) and the ratio *ρ/ρ*_RUC_ is chosen to eliminate the effect of packing mode.

**Fig 13:**
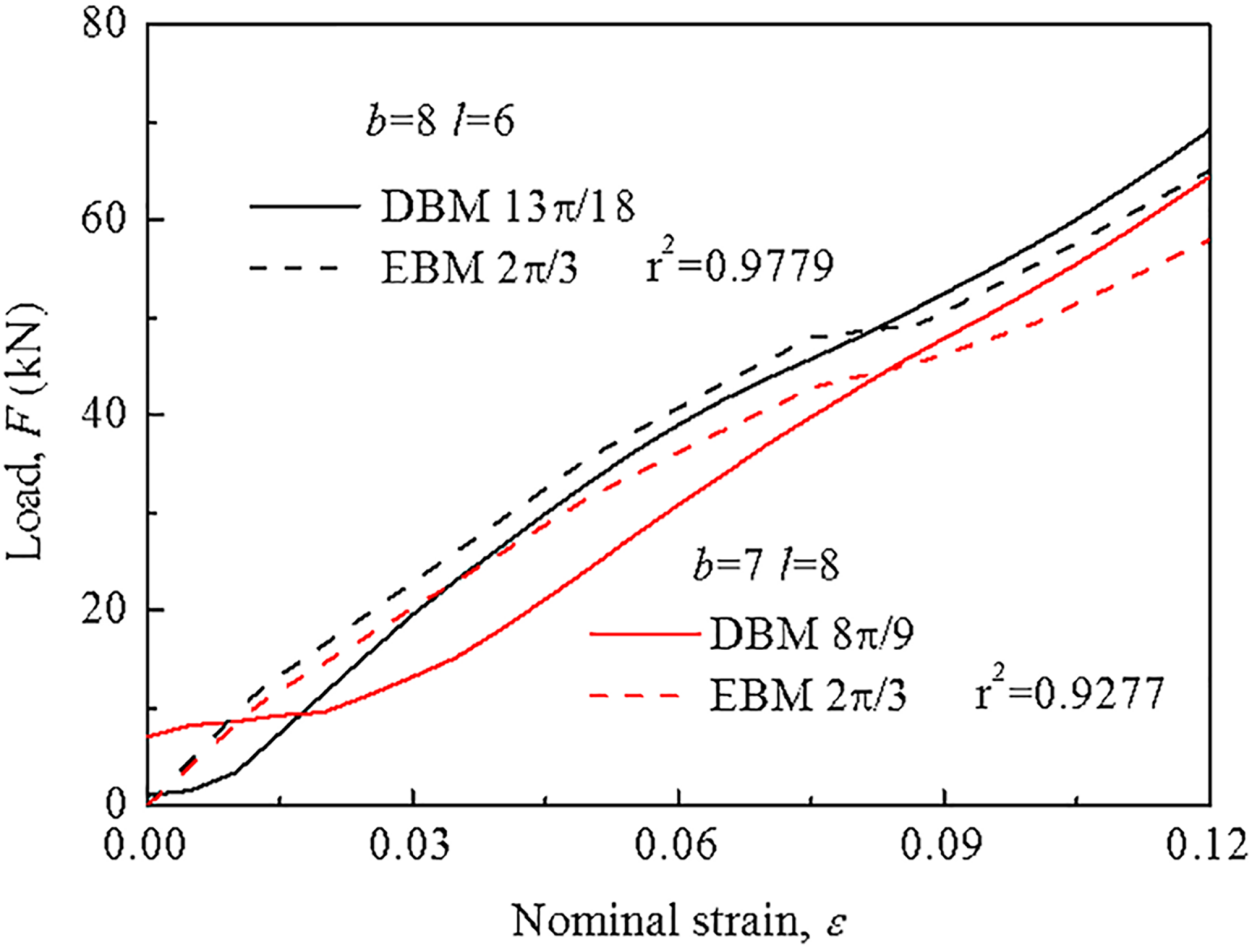
Validation for asymmetrical packing modes. Validation for predicting mechanical behavior of the module neglecting load plateau stage for asymmetrical packing modes by the EBM model for hexagonal dense packing.

To validate this model for asymmetric packing modes, two packing forms were chosen with different sizes, modes, and densities, namely, *θ* = 13*π*/18, where *b* = 8 and *l* = 6, and *θ* = 8*π*/9, where *b* = 7 and *l* = 8. Fig 13 shows the mechanical responses neglecting the load plateau of the module, and it is observed that there is a satisfactory agreement with the EBM model for hexagonal packing mode under the same size (where r^2^ = 0.93–0.98), suggesting the accuracy and capability of the EBM model in predicting the mechanical behavior of the battery module.

## Concluding remarks

Correctly predicting the mechanical behavior of the module subjected to mechanical loading is critical to understanding the mechanical integrity of the battery pack and ensuring full vehicle crash safety. In this paper, a general method to establish the computational homogenized EBM model is proposed. For the convenience of study, we uniformly defined the packing size and mode of the module. Moreover, the RUC was extracted from the module and the periodic boundary condition was applied on it to acquire the homogenized mechanical properties. The experiments and corresponding DBM model were performed to validate the proposed homogenized EBM model under constrained compression loading conditions. For the cubic dense packing mode, the EBM model can well predict the mechanical behavior with length scale up to 15 × 15 and 15% deformation. For the hexagonal dense packing mode, the revised EBM model based on the packing density can represent well the mechanical properties of the module in the range of 12% deformation under different sizes. For asymmetrical packing modes where the structure of the module is unstable, after removing the load plateau stage that was caused by the relative motion of individual cells, the mechanical behavior of the module can be predicted well by the EBM model for hexagonal dense packing with the same size. Therefore, the homogenized modeling method is widely applicable for different packing modes.
